# Characterizing the Norway Lobster *Nephrops norvegicus* (L., Homarida: Decapoda) Maturity of Males in the Mediterranean Sea: Morphological and Physiological Aspects

**DOI:** 10.3390/ani15233478

**Published:** 2025-12-02

**Authors:** Cristina Porcu, Noemi Pascale, Andrea Bellodi, Pierluigi Carbonara, Alessandro Cau, Riccardo Demurtas, Antonello Mulas, Maria Cristina Follesa

**Affiliations:** 1 Department of Life and Environmental Sciences, University of Cagliari, Via T. Fiorelli 1, 09126 Cagliari, Italy; abellodi@unica.it (A.B.); alessandrocau@unica.it (A.C.); ricdemu@hotmail.com (R.D.); antonello.mulas@unica.it (A.M.); follesac@unica.it (M.C.F.); 2 Department of Veterinary Medicine, University of Sassari, Via Vienna 2, 07100 Sassari, Italy; 3 Department of Biomedical Sciences, University of Sassari, Via San Pietro 43/b, 07100 Sassari, Italy; 4 Department of Integrated Marine Ecology, Calabria Marine Center, Stazione Zoologica Anton Dohrn (SZN), C.da Torre Spaccata, Località Torre Spaccata, 87071 Amendolara, Italy; 5 Fondazione COISPA, Via Trulli, 18-20, 70126 Bari, Italy; carbonara@coispa.it; 6 CoNISMa Consorzio Nazionale Interuniversitario per le Scienze Mare, Piazzale Flaminio 9, 00196 Rome, Italy

**Keywords:** *Nephrops norvegicus*, males, size at onset of maturity, histology, morphometric maturity, Mediterranean Sea

## Abstract

In male decapods, such as the Norway lobster *Nephrops norvegicus*, sexual maturity is not easily determined by macroscopic examination of gonads and few studies have been conducted on this topic. Sexual maturity is linked to changes in both external morphology and physiology. This study analyzed the maturity of *N. norvegicus* males in the Central-Western Mediterranean Sea (Sardinia), focusing on primary sexual characteristics (testes and vasa deferentia) and morphometric traits (appendix masculina and petasma). Since the testes showed no macroscopic changes in size during ontogeny, histological analysis was performed. It revealed that spermatogenesis occurred year-round in the Sardinian population, with the smallest mature individual at 18.3 mm carapace length (CL). All individuals above 23.5 mm CL had testes and vasa deferentia filled with spermatozoa and were considered physiologically mature. Morphometric maturity was observed between 27.7 and 36.2 mm CL, based on appendix masculina and petasma measurements. Despite a close association, secondary sexual characteristic development and physiological maturity were not synchronized, as seen in other crustaceans. This discrepancy likely reflects differences in male reproductive strategies, as males may produce spermatophores at smaller sizes (e.g., 23.5 mm CL), though there is no evidence that such males can successfully inseminate females in the wild.

## Simple Summary

This study analyses the maturity for male Norway lobsters in Sardinian waters, focusing on primary sexual characteristics and morphometric traits. The aim was to understand the timing of maturity for better fisheries management. Results revealed that spermatogenesis occurs year-round, with the smallest mature individual at 18.3 mm carapace length (CL). Physiological maturity was reached in males over 23.5 mm CL, while morphometric maturity occurred between 27.7 and 36.2 mm CL, based on appendix masculina and petasma size. The study found that secondary sexual characteristic development and physiological maturity are not always synchronous. These findings have important implications for sustainable management of this commercially exploited species, as they offer insights into reproductive success and population dynamics.

## 1. Introduction

Determining the body size at which individuals first become capable of reproducing (SOM) is essential for understanding how different species allocate energy to reproduction and how this affects their overall reproductive success [1]. Reaching maturity at an earlier stage can be advantageous because it shortens the time needed to produce the next generation and reduces exposure to juvenile mortality. Conversely, organisms with delayed maturation benefit from lower instantaneous rates of juvenile mortality and higher initial and overall lifetime fecundity [1,2]. In many crustaceans’ species, the estimation of size at maturity is based on the analysis of female maturity ogives, a classical method that relies on the direct observation of gonadal development [3,4]. During gonadal maturation, the ovaries undergo a sequence of macroscopic changes in their morphology (mainly in their relative size), which are easily detectable by a naked-eye [5,6,7]. In male decapods, instead, maturity cannot be readily determined from macroscopic examination of gonads and associated structures (e.g., androgenic gland, and vasa deferentia), and few such studies have been carried out (e.g., [1,8,9]). Additionally, in both genders, sexual maturity is associated with alterations in both external morphology and physiology, on which bases, different types of maturity can be defined: physiological, morphometric, and functional [10,11]. Physiological maturity is defined as the capacity of individuals to produce gametes [10]. In crustaceans, this condition is identified by the presence of spermatophores in the vasa deferentia in males and the development and colour of the ovaries in females [1,4,10,12]. On the other hand, allometry in the development of specific body features (i.e., secondary sexual traits) could be used to estimate morphometric maturity [11,13]. Theoretically, this allometry is connected to biochemical and physiological changes that occur throughout sexual maturation, including variable rates of somatic growth and behaviors connected to mating [4,12]. In the reproductive studies, the application of such morphometric analysis has several benefits: it is less time-consuming compared to histological examination of primary sexual characteristics, more economical and practical, since it does not require specialized personnel, laboratory infrastructure, or costly equipment, and it permits to describe the allometric growth changes connected to the maturity aspects [13,14,15]. Finally, the third type of maturity, the functional one, is defined as the ability of individuals to mate successfully and to produce offspring [4,10,12]. This measure requires individuals to be physiologically and morphometrically mature [10,11,12].

The Norway lobster *Nephrops norvegicus*, Linnaeus 1758 (Homarida: Decapoda) is a common decapod crustacean inhabiting the muddy bottoms [16,17] of the European continental shelves and slopes, at depths from 10 to 800 m [17]. In the Mediterranean Sea, the highest densities are found between 200 and 500 m, although in the Adriatic Sea high catch rates are reported at depths from around 30 m to over 400 m with peak of concentrations observed at depths of 70 m off Ancona, 80–90 m in the Velebit Channel, and 200 m in the Jabuka/Pomo pit [15,18,19,20]. It represents one of the most valuable fishing resources in European waters, holding substantial commercial importance throughout its geographical distribution [21,22,23]. Annual global capture production in European waters has remained relatively stable at approximately 60,000 tons over the past three decades [23,24,25,26]. In the Mediterranean Sea, *N. norvegicus* is primarily harvested by bottom trawling, a fishing method that simultaneously targets other demersal commercial species, including the European hake (*Merluccius merluccius*), anglerfish (*Lophius budegassa* and *L. piscatorius*), horned octopus (*Eledone cirrhosa*), and blue whiting (*Micromesistius poutassou*) [21]. This high-prized resource, due to its largely sedentary, benthic lifestyle and the patchy distribution of populations, is vulnerable to local scale depletion [27,28].

*Nephrops norvegicus* is dioecious with visible external organs for reproduction [29,30,31]. As in many commercial species, the female reproductive biology of the Norway lobster has been extensively studied (e.g., [29,32,33,34,35,36,37,38]). However, despite recent findings on the important role of males in maintaining the balance of fished decapod populations [39,40,41,42,43], very little is known about the male biology of *N. norvegicus*. The ovaries mature in spring, when the molting also occurs after the egg-hatching stage. The post-molt period, in late spring, seems to be the only period available for copulation [31]. During copulation, the spermatophore is passed to the female via grooves in the petasma by the action of the appendix masculina (am), a visible rounded body structure located on the endopod (inner side) of the second pair of pleopods which is associated with the sperm transfer [31,44]. It is also known that males with petasma and am ablated cannot copulate [45]. Spermatophores are transferred together with vast amounts of seminal fluids, which harden and form a kind of sealant that occupies most of the space in the thelycum, the females’ sperm-storage organ [9,46].

Since physiological and morphometric maturities are simultaneous events in many crustacean species [47,48], we hypothesized that also the Norway lobster could show a similar pattern. For this reason, the primary aim of this study is to assess male physiological maturity through macroscopic examination of the reproductive system and histological analysis of the testes and vasa deferentia, in order to determine the SOM and provide the first description of the male reproductive period in the Mediterranean Sea. We also provide an extensive morphometric analysis of secondary sexual characteristics, such as am and petasma, related to the carapace length of males to investigate changes in allometric growth associated with maturation. Given the worldwide popularity of the species as seafood [49,50], all these data could contribute to increasing the biological knowledge on *N. norvegicus* in the Central Western Mediterranean, providing important key life-history parameters recognized as essential for fisheries management.

## 2. Materials and Methods

### 2.1. Sampling and Biological Data Collection

**Figure 1:**
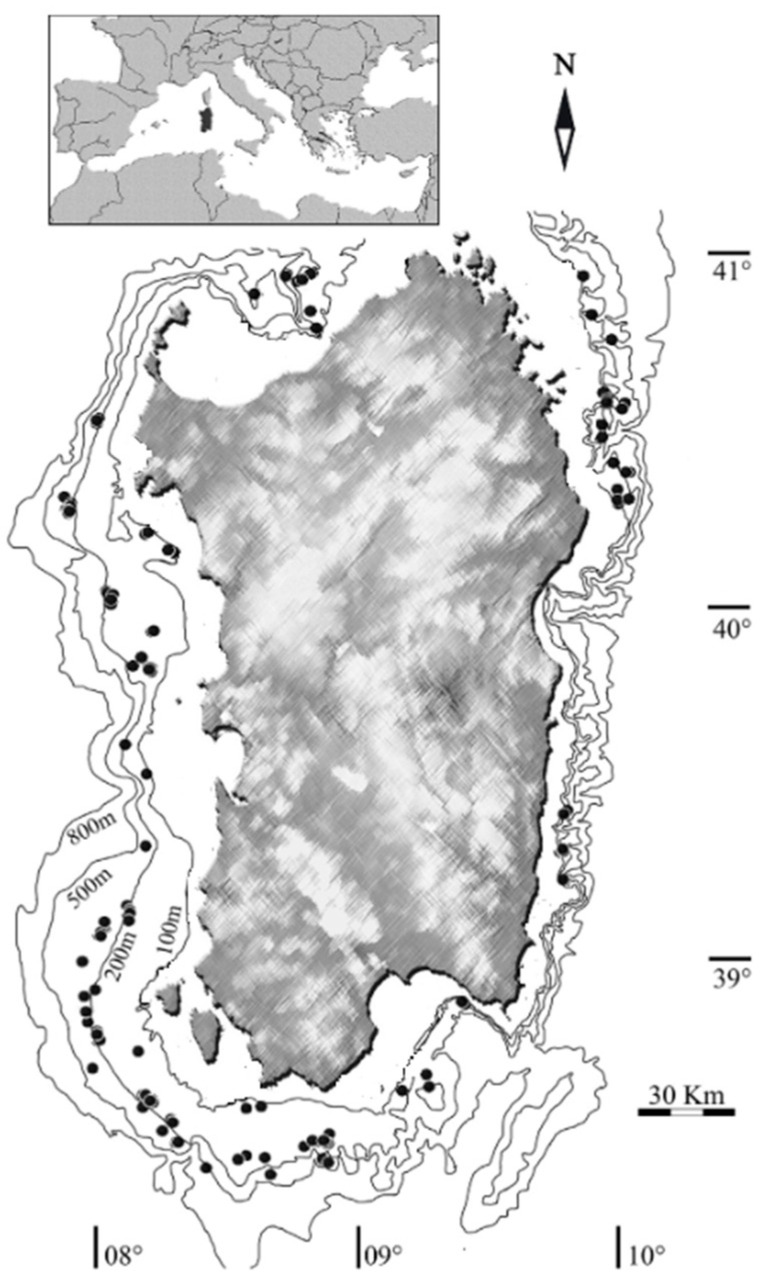
Map of the study area. Black dots represent the hauls where *N. norvegicus* specimens are collected.

All examined male specimens were collected monthly from June 2020 to August 2022 around Sardinian waters (Central Western Mediterranean) (Figure 1) at depths between 321 m and 666 m during the Mediterranean International Trawl Survey (MEDITS) [51] and commercial hauls along with data from commercial landings through the Data Collection Framework (European Union regulation 199/2008). The collection and handling of animals strictly followed the ethical and welfare considerations approved by the ethics committee of the University of Cagliari (Sardinia, Italy).

For each individual, carapace length (CL, mm) to the nearest half mm and sex were recorded. The total mass (TM, g, to the nearest 0.1 g) was recorded only for individuals possessing both claws.

### 2.2. Analysis of Gonado-Somatic Index (GSI%) Evolution Among Sampled Seasons

For a subsample of males, the testes were removed and weighed (TW, 0.01 g) to estimate the Gonado-Somatic Index (GSI%), by size class and season, calculated as follows:

$$GSI\%=\left(\frac{TW}{TM}\right)\times 100$$

Potential significant differences in GSI% evolution were tested using one-way ANOVA [52].

### 2.3. Population Size-Structure and Allometric Growth Equation

The size–frequency distribution of the whole male population was evaluated for each size class (1 mm CL) and the normality of distribution was analized with the Shapiro–Wilk test [53,54].

The allometric growth of the specimens was evaluated by fitting the power equation:

$$TM=a\times {CL}^{b}$$

where TM is total weight, CL is carapace length, *a* the intercept, and *b* the allometric coefficient. Parameters were estimated applying the logarithm-transformed linear model expressed as the following equation:

$$LogTM=\left(loga\right)+b\times \left(logCL\right)$$

where “*a*” represents the intercept of the regression line and “*b*” the slope of the relationship [55].

### 2.4. Macroscopic Observation of Reproductive System and Histological Procedure 

Macroscopical observations of testes and vasa deferentia belonging to males with visible reproductive system, by naked eye, were carried out and one specimen by each size class (5 mm CL) was photographed with a camera CANON 650D (Tokyo, Japan).

A subsample of testes and vasa deferentia belonging to individuals of all size classes, seasonally caught, were removed and fixed for histological procedures. For the specimens below 20 mm CL, the entire abdomen was fixed and the gonads and vasa deferentia removed after 48 h of fixation. The tissues were dehydrated through a series of ascending ethanol solutions (70–100%) (Carlo Erba, Cornaredo, Italy), embedded in a synthetic resin (GMA, Technovit 7100, BioOptica, Milan, Italy) following routine protocols, and sectioned at 3.5 µm with a rotating microtome (ARM3750, Histo-Line Laboratories, Pantigliate, Italy). Slides were stained with hematoxylin and eosin (H&E, BioGnost, Zagreb, Croatia) for standard histology. Subsequently, sections were rapidly rinsed in 96% ethanol and dehydrated in two changes of absolute ethanol for 5 min each, cleared in Histolemon (Carlo Erba Reagents, Cornaredo, Italy) and mounted in resin (Eukitt, Bio-Optica, Milan, Italy). Selected sections were observed and photographed using a Nexcope NE600 optical microscope (Ningbo Yongxin Optics Co., Ltd., Ningbo, Zhejiang Province, China) equipped with a digital camera (MD6iS) (ORMA S.r.l., Sesto San Giovanni, Italy) at different magnifications (40×, 100× and 400×). Macroscopic observations of the testes and vasa deferentia were performed on a subset of males selected from the full pool of 915 individuals. Only specimens in optimal condition, in which the gonads could be clearly exposed and documented after dissection, were included. Because macroscopic integrity varied considerably among individuals, especially in larger specimens, one representative male per 5-mm CL class was photographed to illustrate the general appearance of the gonads.

It must be noted that this subsampling does not capture intra-class variability; therefore, macroscopic observations were used exclusively for illustrative and descriptive purposes rather than for quantitative assessment of maturity within each size class. Although binomial 95% confidence intervals were computed, they were not informative because only one individual per class was suitable for macroscopic assessment (*n* = 1). Consequently, these observations should be interpreted with caution, as they may not accurately reflect the full variability of the population.

### 2.5. Measurements of Secondary Sexual Characteristics

**Figure 2:**
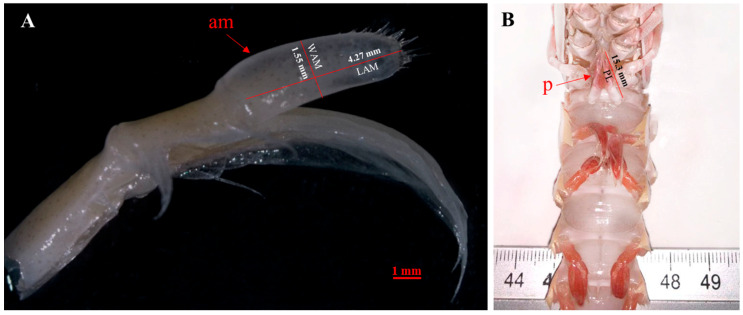
Positions of morphometric measurements analyzed of *N. norvegicus*: (**A**) Appendix masculina (am) length (LAM, mm) and width (WAM, mm); (**B**) petasma (p) length (PL).

Beyond histological analysis of testes and vasa deferentia, the second pair of pleopods was excised at the base to ensure the intact removal of the endopodite and the am. The pleopods were stored in 70% alcohol [1] and a digital image of each am was recorded through a Pixelink microscope camera PL-A686C annexed to a stereomicroscope (Zeiss Stemi 2000-C, Jena, Germany). The length (LAM, mm) and width (WAM, mm) of the am were then measured with TpsDig2 software 2.32 version [56] (Figure 2A). The length of petasma (PL) was also taken with the use of calipers to the nearest 0.01 mm (Figure 2B).

### 2.6. Statistical Analyses

The measurements of am (LAM and WAM) of left and right pleopods were compared and statistically tested with a with Student’s *t*-test.

The relationships between carapace length (CL) and each morphometric variable (LAM, WAM, and PL) were investigated to describe allometric growth patterns. Initially, a generalized linear model (GLM) was fitted for each relationship to evaluate the overall association between variables. Subsequently, a segmented (broken-line) regression model [57,58,59] was applied to detect possible discontinuities in the growth trajectories and to estimate the optimal breakpoint (ψ) corresponding to a significant change in slope. This methodology allows for the identification of one or more breakpoints along a continuous regression, where the slope of the fitted line significantly differs before and after ψ. The breakpoint estimation was performed iteratively using the algorithm implemented in the segmented package in R (RStudio 2024.12.0 Build 467), which minimizes the residual variance between the two fitted linear segments [57]. The statistical significance of the change in slope was assessed using the Davies’ test [60], as recommended for piecewise regression models where the breakpoint is not known a priori [61]. For each fitted model, regression parameters (intercepts and slopes for both segments), coefficients of determination (R^2^), standard errors, and *p*-values were recorded. In this analytical framework, the segmented regression identifies two distinct linear components: (1) a pre-breakpoint segment, representing the group of theoretically immature males, and (2) a post-breakpoint segment, corresponding to individuals displaying morphometric features consistent with maturity. The breakpoint (ψ) thus represents the inflection point at which the relationship between CL and the morphometric variable (LAM, WAM, or PL) changes significantly, and is interpreted as the SOM. The estimation of the inflexion point corresponding to the SOM was investigated using the segmented robust regression analysis, as suggested by [4,15,61]. Although the relationship between CL and morphometric traits is commonly described by an allometric power-law equation (Y = a × CLb; Y = a × CLb; Y = a × CLb), the segmented regression approach does not assume such a functional form a priori. Instead, it provides a flexible statistical framework that allows for the detection of significant changes in slope along a continuous morphometric gradient, thereby identifying shifts in growth rate consistent with the attainment of sexual maturity [57,62,63]. Significant breakpoints could be interpreted as evidence of morphometric allometry and used as proxies for the estimation of SOM in male *N. norvegicus*.

All statistical analyses were performed using RStudio 2024.12.0 Build 467 [64]. Descriptive statistics are expressed as mean ± standard deviation (SD), and the threshold for statistical significance was set at *p* < 0.05. The R packages used in this study are listed in Table 1.

## 3. Results

### 3.1. Gonado-Somatic Index, Populations Size-Structure and Allometric Growth

A total of 915 male specimens were analysed (2020: *n* = 209; 2021: *n* = 345; 2022: *n* = 361; winter: *n* = 222; spring: *n* = 276; summer: *n* = 254; autumn: *n* = 163). The Shapiro–Wilk test confirmed a normal distribution of the data (*p* = 0.79). Among the total amount of collected specimens examined for the population structure analysis, many individuals were missing appendages and therefore could not be accurately weighed. As a result, 448 specimens were used for the size–weight relationship analysis.

Furthermore, both the am and the petasma are external structures that are particularly fragile and prone to breakage or damage. For this reason, 687 specimens could be used for the analyses of the am, whereas only 188 specimens were suitable for the petasma measurements.

**Figure 3:**
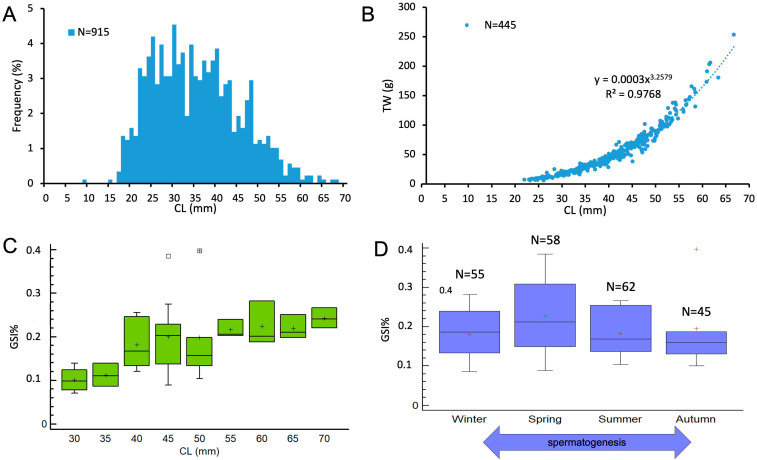
Size–frequency distribution (**A**) and size–weight relationship (**B**) of *N. norvegicus* males around Sardinian seas. Evolution of Gonado-somatic Index (GSI%) of male population of *N. norvegicus* by size classes (**C**) and seasons (**D**). In (**D**), the double-headed arrow indicates the duration of spermatogenesis. The boxes show the interquartile range, with the median value indicated by the horizontal line and the ‘+’ sign indicating the mean; whiskers show the range. Individual symbols show outliers. (Winter, January–March; Spring, April–June; Summer, July–September; Autumn, October–December).

The size–frequency distribution of males revealed a CL ranging from 17.6 to 68.5 mm (mean ± standard deviation [SD] = 34.7 ± 10.1 mm, *n* = 915) (Figure 3A). The length–weight relationship (Figure 3B) showed a strong positive correlation between CL and TW (*R*^2^ = 0.9798), indicating positive allometric growth (*b* > 3).

GSI did not vary significantly among 5 mm CL size classes (ANOVA: *F* = 1.9, *p* = 0.09) (Figure 3C), although a slight increase was observed in the mean gonad weight of males measuring between 30 and 45 mm CL. Similarly, the seasonal variation in GSI was not statistically significant (ANOVA: *F* = 0.69, *p* = 0.56), suggesting no marked temporal pattern in male reproductive activity during the sampling period.

### 3.2. Primary Sexual Characteristics Analysis

#### General Anatomy of the Male Reproductive System

**Figure 4:**
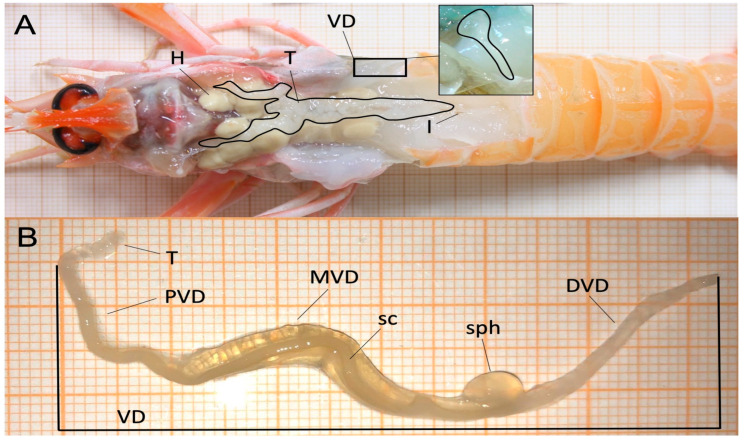
(**A**) Dorsal image of a *N. norvegicus* male showing the hepatopancreas (H), the testes (T), the intestine (I) and the vasa deferentia (VD). The black box indicates the VDvasa deferentia in detail. (**B**) High magnification of VD, which is divided into three distinct regions: the proximal VD (PVD), the middle VD (MVD) in which the sperm cord (sc) is stored and that enlarges at the end to form the sphincter (sph) and distal VD (DVD).

The internal reproductive system of *N. norvegicus* males is mainly composed of paired testes and vasa deferentia. Testes are a thin, semitransparent or slightly yellowish organ that run parallel to the median plane of the body. They are located under the carapace (in the picture partially removed to show the inner side of the animal) (Figure 4A) and have a double H-shape, that extend to the dorso-lateral stomach and up to the base of the eyestalks, with two shorter posterior lobes that extend through the abdomen to the intestine. The pair of vasa deferentia is semi-transparent like the testes and arises from each side of the double H, extending ventrally to an opening pore (the gonopore) at the coxa of the fifth walking leg (Figure 4B). Each vasa deferentia has a sphincter and is divided in three distinct portions, based on morphological and functional criteria (proximal, middle and distal, Figure 4B).

**Figure 5:**
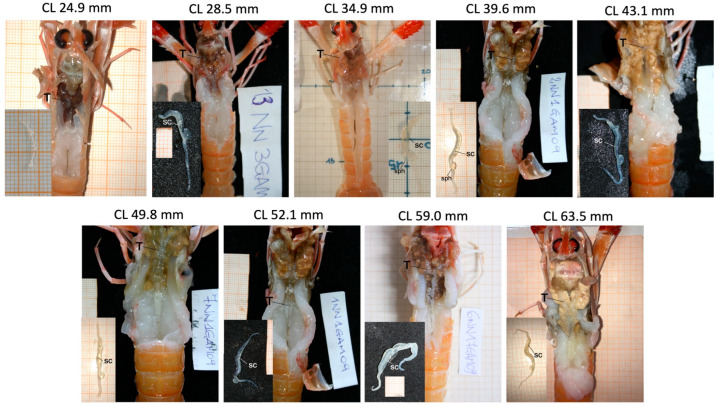
Gross morphology of the reproductive system of *N. norvegicus* males in which testes and vasa deferentia have the same appearance in each 5 cm size-class. For each individual, a detailed image of vasa deferentia is reported. CL, carapace length; sc, sperm cord; sph, sphincter; T, testes.

As illustrated in Figure 5, the testes retained a consistent morphology and overall structure in the photographed specimens across size classes. In the limited subset of individuals suitable for macroscopic examination, the testes and vasa deferentia generally appeared well developed, and a whitish line corresponding to the sperm cord was visible in several cases (Figure 5). Because only one representative male per 5-mm CL class could be photographed, these observations are descriptive and cannot provide quantitative information on the prevalence of maturity within each size class. They should therefore be interpreted with caution and viewed as illustrative rather than representative of the full population variability.

### 3.3. Histology

A total of 53 males with CL ranging from 17.6 to 67.7 mm (mean ± standard deviation [SD] = 29.09 ± 10.57 mm), caught in all seasons, was histologically examined.

**Figure 6:**
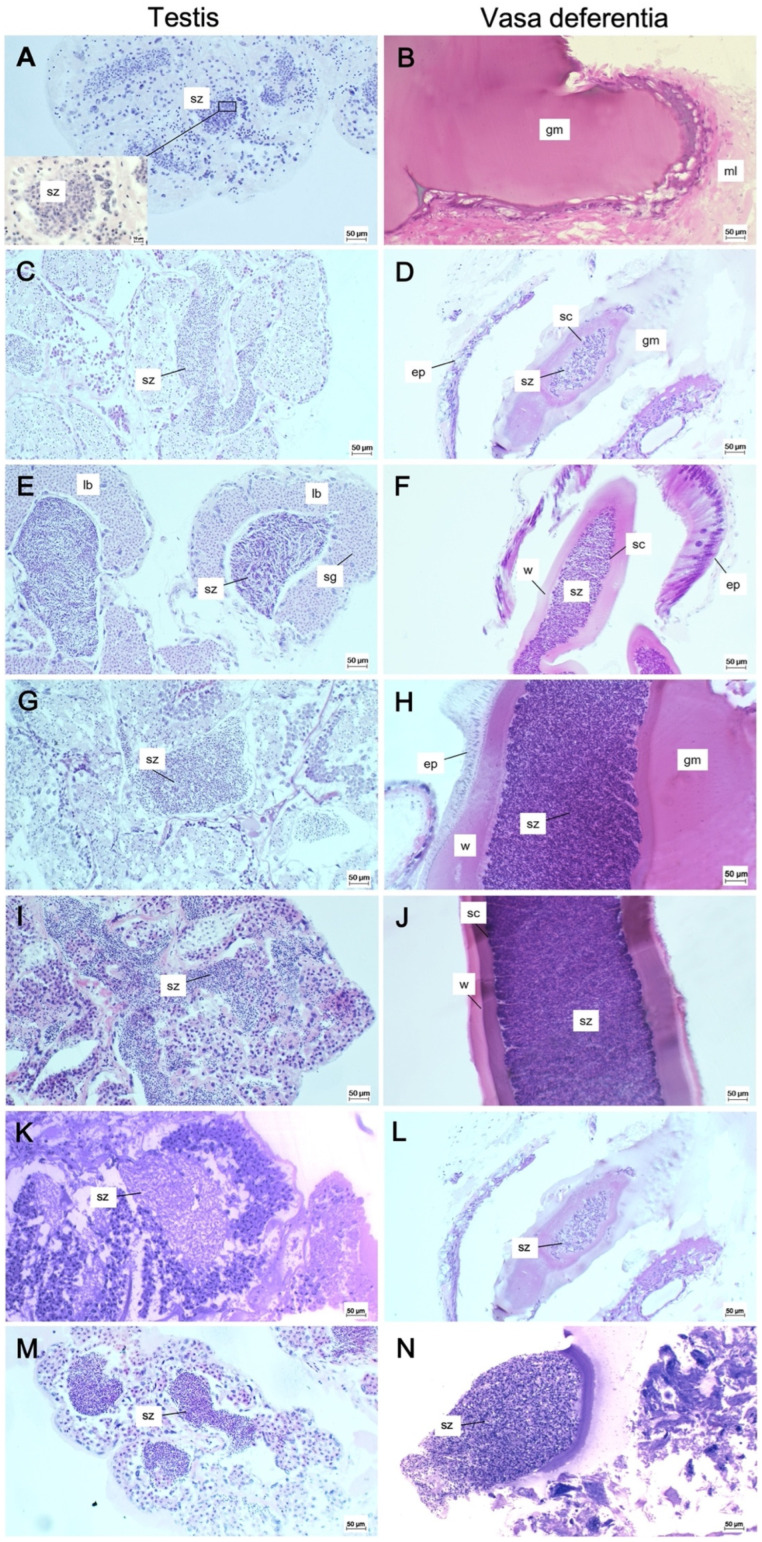
Histological sections of testes (**A**,**C**,**E**,**G**,**I**,**K**,**M**) and vasa deferentia (**B**,**D**,**F**,**H**,**J**,**L**,**N**) of *N. norvegicus* males at different size classes. (**A**) Transverse section of testis in which mass of spermatozoa is visible also in detail (CL 18.3 mm, May). (**B**) Longitudinal section of an empty vasa deferentia where only an amorphous gelatinous mass (CL 18.3 mm, May) is present. (**C**) Mature testis in which spermatozoa are densely packed inside the lobule (CL 23.5 mm, February). (**D**) Longitudinal section of vasa deferentia where the spermatozoa are packed in the lumen and the unique sperm cord is formed (CL 23.5 mm, February). (**E**) Transverse section of testis where the spermatogonia are in the periphery and the spermatozoa are in the centre of the lobule (CL 30.0 mm, February). (**F**) Longitudinal section of vasa deferentia in which the wall of the sperm cord is made by the secretion of the cubic epithelium (CL 30.0 mm, February). (**G**) Mature testis where spermatozoa are packed inside the lobule and the spermatocytes are in the periphery (CL 50.9 mm, March). (**H**) Vasa deferentia where the single sperm cord is maintained by the gelatinous mass (CL 50.9 mm, March). (**I**) Active spermatogenesis of a mature testis (CL 58.6 mm, June). (**J**) Longitudinal section of vasa deferentia with densely packed spermatozoa (CL 58.6 mm, June). (**K**) Mature testis with mature spermatozoa (CL 28.6 mm, August). (**L**) Longitudinal section of vasa deferentia with densely packed spermatozoa (CL 28.6 mm, August). (**M**) Mature testis in which spermatozoa are densely packed inside the lobule (CL 43.4 mm, November). (**N**) Vasa deferentia with densely packed spermatozoa (CL 43.4 mm, November). ep, epithelium; gm, gelatinous mass; lb, lobule; ml, muscle layer; sc, sperm cord; sz, spermatozoa; w, wall.

Microscopically, the testes consist of several lobules interconnected by connective tissue toward the lumen. The lobular testes are surrounded by a thin wall, formed by a germinal epithelium, which lies above a basal lamina covered by two thin layers: one of connective tissue and one of muscle layer. The testes are, overall, organized in many lobules, each with an independent production cycle, opening into sinuous collecting tubules that transport the developing sperm to the vasa deferentia (Figure 6), through which secondary spermatophores are formed.

Histological sections of testes and vasa deferentia of *N. norvegicus* males during the present investigation indicate that spermatogenesis occurs throughout the year, and that spermatophores are always carried in the vasa deferentia. The smallest individual with fully developed spermatozoa in the tubules of the testes has 18.3 mm CL, but empty vasa deferentia (Figure 6A,B). Therefore, sexual maturity occurs at a CL of 23.5 mm (Figure 6C,D), the smallest size in which a single sperm cord of spermatozoa is found in the vasa deferentia. All analysed individuals, greater than 23.5 mm CL, showed mature testes and vasa deferentia full of spermatozoa (Figure 6E–N).

### 3.4. Statistical Analysis

#### Segmented Regression Analysis

A total of 687 am were collected and analysed to investigate the segmented relationships between CL and am measurements. No significant differences were detected between the left and right pleopods (LAM: *t*-test, *p* = 0.81; WAM: *t*-test, *p* = 0.75); therefore, only one measurement per individual was retained for subsequent analyses.

In addition, a total of 188 PL was measured, as several specimens exhibited damaged or poorly preserved structures due to the fragile nature of this external reproductive appendage.

**Figure 7:**
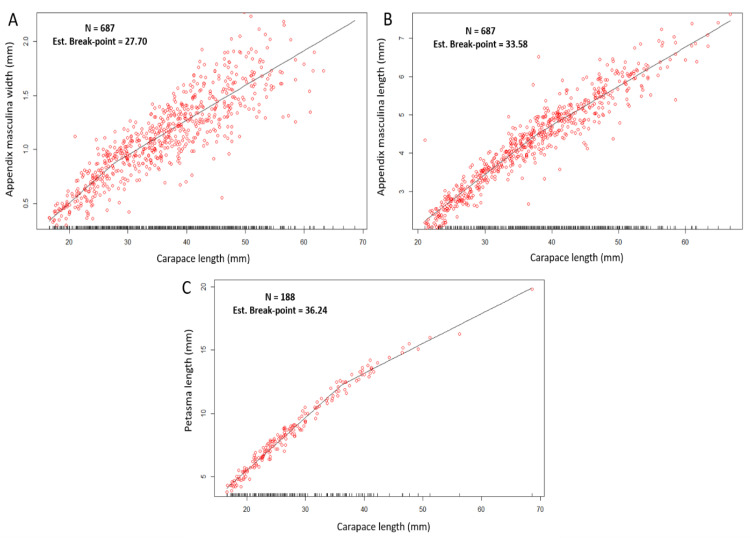
Segmented regression between the variables carapace length (mm) and appendix masculina width (mm) (**A**), carapace length and appendix masculine length (mm) (**B**), carapace length (mm) and petasma length (mm) (**C**). N, number of samples; Est. break-point, Estimated break-point. The R^2^ of the segmented regressions are given in Table 2.

The CL at which males appeared to attain morphometric maturity ranged from 27.7 mm (Figure 7A) to 33.58 mm (Figure 7B), corresponding to the breakpoints estimated for WAM and LAM, respectively.

The analysis of the petasma length (PL) indicated a higher breakpoint, estimated at 36.24 mm CL (Figure 7C).

All the segmented regression models for the am and p measurements yielded statistically significant results, as confirmed by the Davies’ test (*p* < 0.05) (Table 2).

Information about SOM of *N. norvegicus* males estimated with different methodologies in different geographical regions are summarized in Table 3. 

## 4. Discussions

Understanding the life-history traits of a species requires detailed knowledge of key processes such as the development of sexual maturity, associated changes in allometric growth, and the age or size at which these transitions occur. In crustaceans, the onset of maturity involves a series of morphological, physiological and behavioral modifications that enable individuals to produce gametes and successfully copulate [68]. Sexual maturity in decapods is often evaluated by linking gonadal development to body size [9,69]. However, while female maturity is easily detectable macroscopically through marked changes in ovarian size and pigmentation, male maturity is far less evident at the macroscopic level. As a result, relatively few studies have fully characterised maturity stages in male decapods [8,70,71,72].

This study represents the first attempt of estimating the SOM of *N. norvegicus* males in the Mediterranean Sea by analyzing the microarchitecture of the testes and vasa deferentia and comparing it to secondary sexual morphometric characteristics. The macroscopic examination of testes in the limited number of individuals suitable for this analysis did not reveal evident changes in overall dimensions across the size range considered. Although a whitish line corresponding to the sperm cord was visible in several specimens, these observations should be regarded as descriptive only, given the restricted number of individuals examined per size class. Therefore, they cannot be considered quantitatively representative of the entire population. This interpretation is further supported by the Gonado-Somatic Index, which did not show significant variation across size classes.

The microstructure of the testes of the Norway lobster followed the general pattern described for astacids [70]. They were composed of numerous seminiferous lobules connected by a seminiferous duct [71]. *Nephrops norvegicus* exhibited an asynchronous maturation pattern of the testes (i.e., each testicular lobule shows a stage of spermatogenesis that is independent by the stages of the adjacent lobules), a pattern similar to other Nephropidae species such as *Homarus americanus* or Astacidea (e.g., [71,72,73] and Caridea species [74]). Crustacean species with asynchronous testes generally showed continuous spermatogenesis [74], as confirmed by our results. Despite the limited number of specimens examined at the microscopic level, all individuals analysed in the present study exhibited characteristics indicative of physiological maturity, including testes containing developed spermatozoa in the lumen of lobules and ejaculate inside vasa deferentia observed in every season, without discernible seasonal variation in spermatic activity. This process, reported here for the first time within the Mediterranean range, appears to be consistent with the observations presented in the Irish Sea population, where spermatophores were carried in the vasa deferentia at all times [1]. In Portuguese waters, instead, spermatogenesis occurred throughout the year, but spermatozoa did not start to build up in the testis’ tubules until July [75]. The smallest captured male showing fully developed spermatozoa in the testes measured 18.3 mm CL, a value consistent with observations reported for the Irish Sea population [30], but differs from [65] in the North Sea, who found a SOM at 30 mm CL (Table 3). In contrast to [1], who describe a size of 15 mm CL in the Irish Sea, in the current investigation, the smallest male with ejaculates (sperm cord) in the vasa deferentia, discernible as a white line and validated by the histological technique, was 23.5 mm CL. *Nephrops norvegicus* individuals from the Sardinian fishing grounds were typically caught by trawl hauls (commercial hauls: 50 mm diamond-mesh size or 40 mm square-meshed; MEDITS hauls: square-mesh of 40 mm or diamond-mesh of 20 mm opening), which most likely did not allow the capture individuals as small as 12 mm CL, as reported by [1], who used a small mesh beam trawl.

The study on growth performance revealed that *N. norvegicus* males had a positive growth allometry based on the deviation of regression coefficient b from 3 (>3). This is in agreement with two recent studies conducted in the Central Mediterranean Sea in which males showed a positive trend in allometric growth [15,76]. Allometry in the growth of different body parts in crustaceans has been linked to changes in the physiology and biochemistry of individuals that occur as part of the sexual maturation process. In this context, males would devote most of their energy to somatic growth in order to improve their chances of territorial defense and mating, whereas females would prioritize reproductive outputs [4,13,77,78].

Studies on decapod crustaceans indicated that the appendix masculina, along with petasma, plays a key role in spermatophore transfer from male to female and for copulation [79,80,81,82,83]. These may serve to adjust the position of a male genital papilla relative to the aperture of a spermatheca, to stimulate the female and act as sensory devices providing information to the male [45]. Some decapod species, such as *Aristaeomorpha foliacea* and *Aristeus antennatus,* are considered mature if they have joined hemipetasmatas, and contain spermatophores in the terminal ampullae of the vasa deferentia [84,85]. In the Norway lobster, such sexual characteristics are not macroscopically detectable and morphometric measurements are required to determine functional maturity. In the present study, appendix masculina length and width, as well as petasma length, were used as morphometric parameters for *N. norvegicus* males to estimate the functional maturity. The three break-points estimated in the present study appear to be noticeably lower than those reported by [15], who applied the same method (segmented regression) for the first time for the Mediterranean Sea (Northern–Central Adriatic Sea, “off Ancona”). According to the results of the present manuscript, *N. norvegicus* males may reach morphometric maturity at smaller sizes in Sardinian waters than in the Northern–Central Adriatic Sea, despite the similar population size-structure reported for both areas (17.6–68.5 cf. 17–75 mm CL) [15]. Conversely, the breakpoint estimates obtained in the present work are consistently higher than those reported for the fisheries-restricted Pomo/Jabuka Pit, a well-known nursery and spawning ground in the Adriatic Sea along Croatian coasts (Table 2) [15]. The comparatively lower breakpoint values observed in Pomo/Jabuka may possibly be influenced by the high local densities of small individuals, which are known to reduce growth rates through density-dependent mechanisms [86,87,88,89,90,91]. Furthermore, the comparison of Sardinian and NE Atlantic break-point values revealed substantial differences (Table 2). Part of this variability may derive from the different statistical methodologies employed across studies, such as reduced major axis regression [1,67] versus ordinary least squares regression [1], which may introduce methodological bias and hinder direct comparability [4]. However, it is possible that all these variations recorded may reflect subtle density-dependent differences brought about differential fishing pressures [1]. Density-dependent effects on growth have been widely documented in *N. norvegicus* and in other lobster species [1,34,92,93], with individuals inhabiting high-density areas generally maturing at smaller sizes than those from less crowded regions. This pattern has been reported in Scottish waters [4,34], in the Northern–Central Adriatic Sea [15], and when comparing the high-density Pomo/Jabuka population with the more heavily exploited “off Ancona” area [94,95,96] where higher break-point values have been recorded [15]. Within this context, the breakpoint values estimated in the present manuscript, intermediate between those of the protected and overexploited Adriatic zones, suggest that the *N. norvegicus* population in Sardinian waters is fully exploited but maintains relatively stable density trends over time [97].

According to our findings, even though secondary sexual character development and physiological maturity are closely related in *N. norvegicus* males, these events were not synchronous. This apparent anomaly is most likely due to differences in male reproductive strategies, as males may be capable of producing spermatophores at relatively small sizes (e.g., 23.5 mm CL), although there is currently no evidence that such small individuals are able to inseminate females successfully in the wild.

Male reproductive success may be enhanced further when the appendix masculina reaches optimal size related to body size for transferring spermatophores to female thelycum. Asynchrony, in which physiological maturity precedes morphological maturity, was also verified in other male crustaceans as *Arenaeus cribrarius* [47], *Libinia spinosa* by [48], *Liocarcinus holsatus* as found by [98], *Scylla serrata* by [99], and *Necora puber* by [100].

This study provides the baseline for studying the maturity of *N. norvegicus* male populations in the Mediterranean Sea by applying two different approaches (histological and morphometrical), which may have potential implications for the management of such an exploited species as the Norway lobster. SOM is, in fact, a critical parameter in stock assessment of commercially harvested fish stocks as it forms the basis of estimating the spawning stock biomass [11]. The relationship between SOM and other metrics might also contribute to improvements in stock assessment and management of the *Nephrops* resource, and potentially be also useful in calibrating the more routinely used estimates. 

## 5. Conclusions

In conclusion, this study provides valuable insights into the sexual maturity of *Nephrops norvegicus* males in the Mediterranean Sea, highlighting the importance of both histological and morphometric approaches for accurate maturity assessment. Additionally, we found that physiological maturity and the development of secondary sexual characteristics are not synchronized, a pattern that may influence reproductive dynamics and has important implications for the sustainable management of Norway lobster populations. These findings are therefore crucial for refining resource assessment methods and for optimizing fisheries management strategies aimed at long-term sustainability.

## Figures and Tables

**Table 1:** List of R-Studio Packages used in the study (RStudio 2024.12.0 Build 467).

Category	Package	Function/Use
Base statistical environment	stats (base package)	Core functions for linear models (lm()), ANOVA (aov()), *t*-tests (t.test()), correlation, and descriptive statistics
Segmented regression	segmented	Estimation of breakpoints and segmented (piecewise) regression models; includes the Davies’ test for slope change significance
Regression diagnostic and ANOVA validation	car	Levene’s test for homogeneity of variance (leveneTest()), and type-II/III ANOVA (Anova())
Robust and generalized regression	MASS	Robust linear models (rlm()), generalized linear models, and support for diagnostic statistics
Data manipulation	dplyr	Data filtering, transformation, and summarization for morphometric and biometric datasets
Data visualization	ggplot2	Graphical visualization of morphometric relationships, segmented regressions, and descriptive plots
Descriptive statistics	psych	Summary and descriptive functions for biological and morphometric variables

**Table 2:** Summary of segmented regression analysis between carapace length (CL, mm) and secondary sexual characteristics of *Nephrops norvegicus* males: appendix masculina width (WAM), appendix masculina length (LAM), and petasma length (PL). For each relationship, the total number of observations (*n*), carapace length range with mean and standard deviation (CL range (mm) (mean ± SD)), estimated breakpoint ± its standard error (BP ± SE), intercept and slope before (Intercept and Slope before BP) and after the break-point (Intercept and slope after BP), R squared values (R^2^) and Davies’ test results for significance in the slope change (Davies’ test (*p*)) are reported. *** indicates a highly significant difference (*p* < 0.001).

Relationship	*n*	CL Range (mm) (Mean ± SD)	BP (±SE) (mm CL)	Intercept	Slope (Before BP)	Intercept	Slope (After BP)	*R* ^2^	Davies’ Test (*p*)
WAM–CL	687	16.6–68.6 (36.3 ± 10.12)	27.70 ± 2.09	–0.45	0.065	–1.63	0.146	0.88	0.0049 ***
LAM–CL	687	16.6–68.6 (36.3 ± 10.12)	33.58 ± 0.93	–1.32	0.218	–7.37	0.402	0.92	<2.2 × 10^−16^ ***
PL–CL	188	16.6–68.6 (27.9 ± 8.36)	36.24 ± 0.69	–3.87	0.295	–9.30	0.621	0.91	<2.2 × 10^−16^ ***

**Table 3:** Summary of information on *N. norvegicus* size at onset of maturity (SOM) estimated with different methodologies in different geographical regions. CL, carapace length; CrL, crusher length; LAM, length of appendix masculina; LSR, least squares regression; MATURE, MATURE 2 regression; PL, length of petasma; RMA, reduced major axis regression; S, segmented regression; T, testes; VD, vasa deferentia; WAM, width of appendix masculina.

Geographical Area	SOM (CL, mm)	Method	Reference
NE Atlantic Ocean			
North Shields area (North Sea)	30.0	Histology (VD)	[65]
Irish Sea	26.5	Allometry (LAM)	[66]
W Irish Sea	15.1	Histology (VD)	[1]
	25.5–26.3 (MATURE); 24.3–26.9 (RMA)	Allometry (LAM)	
Clyde (Scottish waters)	27.48 (S)	Allometry (LAM)	[4]
Fladen (Scottish waters)	41.12 (S)		
North Minch (Scottish waters)	31.88 (S)		
Noup (Scottish waters)	43.2 (S)		
Sound of Jura (Scottish waters)	22.77 (S)		
Stanton Banks (Scottish waters)	36.04 (S)		
Moray Firth (Scottish waters)	30.14 (S)		
Firth of Clyde (Scottish waters)	29.0–46.3 (LSR); 24.0–46.3 (RMA)	Allometry (CrL)	[67]
Mediterranean Sea			
Off Ancona (Italy, N Adriatic)	41.67 (LAM, S); 42.82 (WAM, S)	Allometry (LAM, WAM)	[15]
Pomo/Jabuk Pits (Croatia, N Adriatic)	24.33 (LAM, S); 25.39 (WAM, S)	Allometry (LAM, WAM)	[15]
Sardinian seas (Italy)	23.5	Histology (VD)	Present study
	18.3	Histology (T)	
	33.53 (LAM, S)	Allometry (LAM, WAM, PL)	
	27.7 (WAM, S)		
	36.24 (PL, S)		

## Data Availability

The original contributions presented in this study are included in the article. Further inquiries can be directed to the corresponding author.
